# A Novel Claudinopathy Based on Claudin-10 Mutations

**DOI:** 10.3390/ijms20215396

**Published:** 2019-10-30

**Authors:** Susanne Milatz

**Affiliations:** Institute of Physiology, Kiel University, Christian-Albrechts-Platz 4, 24118 Kiel, Germany

**Keywords:** tight junction, paracellular permeability, paracellular sodium transport, thick ascending limb, nephropathy, HELIX syndrome, hypokalemia, hypermagnesemia, anhidrosis, gland dysfunction

## Abstract

Claudins are key components of the tight junction, sealing the paracellular cleft or composing size-, charge- and water-selective paracellular channels. Claudin-10 occurs in two major isoforms, claudin-10a and claudin-10b, which constitute paracellular anion or cation channels, respectively. For several years after the discovery of claudin-10, its functional relevance in men has remained elusive. Within the past two years, several studies appeared, describing patients with different pathogenic variants of the *CLDN10* gene. Patients presented with dysfunction of kidney, exocrine glands and skin. This review summarizes and compares the recently published studies reporting on a novel autosomal-recessive disorder based on claudin-10 mutations.

## 1. Introduction

### 1.1. Claudin-10

The protein family of claudins is a key component of the tight junction (TJ). Claudins comprise four transmembrane segments (TM1-4), two extracellular segments (ECS1 and 2) and intracellular N- and C-termini. Embedded in the plasma membranes of adjacent cells, they interact with each other within the same plasma membrane but also across the paracellular cleft, with claudins of the neighboring cell (cis- or trans-interaction, respectively). By this means, they form a complex strand meshwork and determine tightness and selectivity of the bicellular TJ. Whereas most claudins exhibit a mainly sealing function, some claudins form size-, charge- and water-selective channels through the TJ [1,2,3,4,5,6,7,8,9,10,11,12,13,14,15,16,17,18].

**Figure 1:**
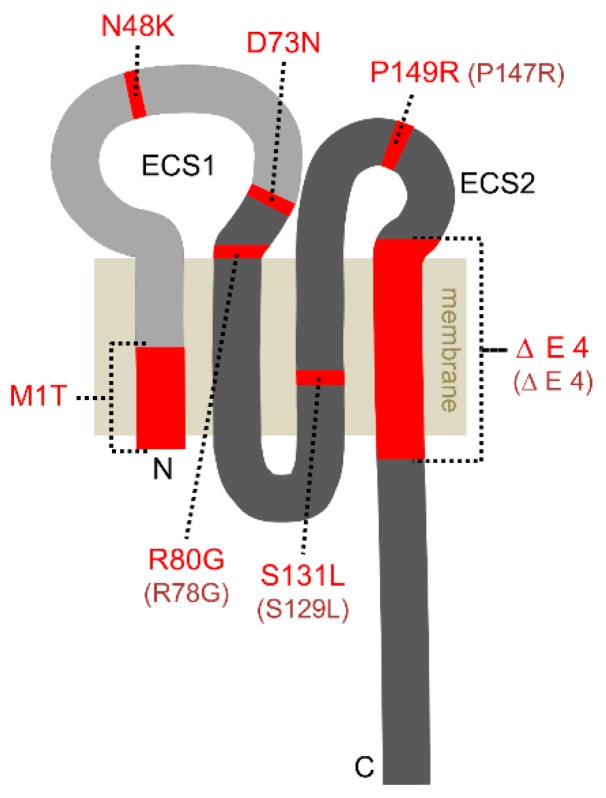
Predicted topology of claudin-10. The two major isoforms claudin-10a and claudin-10b differ in their first transmembrane segment and most of the first extracellular segment (ECS1), both shown in light grey. The remaining protein sequence is identical (shown in dark grey). The mutations discovered to date (red) comprise single amino acid substitutions or large deletions (M1T, ΔE4) and affect either both claudin-10a and claudin-10b or only claudin-10b. The existing claudin-10a variants with respective residue numbering are depicted in parentheses.

The claudin family encompasses at least 24 members in mammals. The human gene encoding claudin-10 (*CLDN10*) contains six exons and gives rise to two major isoforms: claudin-10a and -10b. According to their usage of either exon 1a or 1b, they differ in their TM1 and ECS1 (Figure 1). As ECS1 acts as main determinant of charge selectivity, claudin-10a and -10b strands exhibit contrarian permeability properties.

Due to claudin-10a’s equipment with seven positive and only one negative amino acid in ECS1, it is predestined to form a paracellular anion channel [10,12]. Expression of human claudin-10a in the poorly ion permeable cell line MDCK C7 resulted in a decrease in transepithelial resistance (TER) without alteration in preference for Na^+^ or Cl^–^ [12]. Moreover, claudin-10a expression increased the relative NO_3−_ permeability but decreased the permeability to the anion pyruvate, suggesting that claudin-10a modifies the anion preference of the paracellular pathway [12].

ECS1 of claudin-10b comprises four positive and five negative amino acids. In most cell culture models, heterologous expression of human or murine claudin-10b led to a marked decrease in TER that was based on an increase in Na^+^ permeability over Cl^–^ permeability (P_Na_^+^/P_Cl_^–^). Further studies revealed a relative strong permeability to all monovalent cations with preference for Na^+^, a lesser permeability to divalent cations and impermeability to larger molecules (4 kDa dextran) or water of the claudin-10b-based paracellular channel [10,12,13,19].

Claudin-10a and -10b do not only differ significantly in their function but also with respect to their expression in the body. Whereas claudin-10a appears to be restricted to the kidney, claudin-10b has been detected in many tissues, including kidney, skin, salivary glands, sweat glands, brain, lung and pancreas [10,12,20]. Along the kidney tubule, claudin-10a is expressed in the proximal tubule to the S3 segment whereas the main expression site of claudin-10b is the thick ascending limb of Henle’s loop (TAL). Claudin-10b is found along the whole medullary–cortical axis of TAL from inner stripe of outer medulla (ISOM) to outer stripe of outer medulla (OSOM) to the renal cortex. In ISOM TAL, to current knowledge, solely claudin-10b constitutes the bicellular TJ, where it facilitates Na^+^ reabsorption [21,22]. Towards OSOM and cortex, a TAL mosaic claudin expression is found. Claudin-10b equips part of the TJs, whereas the remaining TJs contain a complex of claudin-3, -16, -19 and to a smaller extent claudin-14 [21,22,23]. This complex is involved in the reabsorption of divalent cations such as Ca^2+^ and Mg^2+^ in the TAL. The thin limb of Henle also incorporates claudin-10, as yet the identity of the present isoform is unknown [24].

An important insight into the physiological role of claudin-10b was provided by the mouse model generated by Breiderhoff et al., lacking claudin-10 in the entire loop of Henle. These mice featured a strongly reduced paracellular Na^+^ selectivity in the TAL that led to a urinary concentration defect and was accompanied by hypermagnesemia, polyuria, polydipsia, elevated plasma urea levels and compensatorily increased K^+^ and H^+^ secretion [25,26]. This misbalanced TAL electrolyte handling was accompanied by a severe medullary nephrocalcinosis in these animals.

### 1.2. Claudinopathies

So far, a number of human hereditary diseases based on defects in claudin-1, -14, -16 and -19 have been reported [27,28,29,30,31,32]. However, for 18 years after the discovery of claudin-10, its functional relevance in men has remained unclear. On the one hand, defects in the *CLDN10* gene are rare and clinical manifestations occur mainly in patients with biallelic defects (autosomal recessive disorder). On the other hand, some patients presented with symptoms many years ago but were originally misdiagnosed with Bartter syndrome or Gitelman syndrome. Both diseases are characterized by a salt-losing nephropathy with an imbalance in Ca^2+^ and Mg^2+^ homeostasis. In Bartter syndrome, the transcellular NaCl reabsorption in the TAL is disrupted, due to mutations in the Na-K-Cl cotransporter 2 (NKCC2), the renal outer medullary potassium channel (ROMK1) or the Cl^–^ channel Kb (ClC-Kb) [33,34,35,36]. Gitelman syndrome is caused by mutations affecting the Na^+^-Cl^–^ cotransporter (NCC) in the distal convoluted tubule [37]. Nowadays, whole-exome sequencing is available at lower cost and increasingly used to identify the cause of rare mendelian disorders. In 2017, three studies reported on patients with different pathogenic variants of *CLDN10* [38,39,40]. Hadj-Rabia et al. coined a novel disease syndrome, summarizing the clinical manifestations of their patients (HELIX for hypohidrosis, electrolyte imbalance, lacrimal gland dysfunction, ichthyosis, xerostomia) [39]. This review aims to summarize and compare the data of Bongers et al., Hadj-Rabia et al., Klar et al. and a case report by Meyers et al., all describing a novel claudinopathy based on *CLDN10* mutations [38,39,40,41].

## 2. Clinical Manifestations

To date, a total of 22 patients carrying homozygous or compound heterozygous *CLDN10* mutations have been reported, their ages ranging from 4 to 53 years. Patients were mostly born in consanguineous families and first symptoms often occurred in early childhood, sometimes directly after birth. Patients presented with first symptoms as anhidrosis, xerostomia, alacrima, muscle cramps, falls, or chest pain. Table 1 provides a summary of all patient groups and their clinical manifestations.

### 2.1. Hypohidrosis, Xerostomia and Alacrima

Hypohidrosis with intolerance to heat was frequently reported as one of the first symptoms observed in early childhood. Apparently, all known patients suffer from hypohidrosis, including the two patients described by Bongers et al., who did not complain about reduced sweating at the outset but confirmed hypohidrosis subsequently [38], (personal communication with Tom Nijenhuis, Radboud University Medical Center, Nijmegen). Klar et al. evaluated heat intolerance in two patients by exposure to heat for 20 min, which resulted in a rapidly increased body temperature from 37 °C to 39.6 °C and an increase in heart rate [40]. Generalized hypohidrosis was verified using starch-iod test applied on different body parts, corroborating a severe dysfunction of eccrine sweat glands.

Likewise, xerostomia due to reduced saliva production is apparently a typical symptom of *CLDN10* defects as it has been documented in all known patients including the patients examined by Bongers et al. [39,40,41]; (personal communication with Tom Nijenhuis). Hadj-Rabia et al. analyzed xerostomia by saliva secretion rate measurements in three adult patients [39]. As a result, the flow of fluid was reduced by 98% in patients compared to healthy controls. Moreover, the fluid/mucus ratio of saliva was dramatically decreased in patients with *CLDN10* variants. Hadj-Rabia also documented a poor dental condition with severe enamel wear and generalized gingival inflammation of their patients [39]. This is attributed to aptyalism but might also be a consequence of disturbed enamel mineralization (amelogenesis imperfecta) as claudin-10 expression was found in ameloblasts of mice [42].

Alacrima (the inability to produce tears) was described in the majority of patients and was confirmed using Schirmer’s test by Hadj-Rabia et al. [39,40,41].

### 2.2. Dermatological Manifestations in Addition to Hypohidrosis

The occurrence of ichthyosis and other dermatological manifestations among patients was rather inconsistent. Hadj-Rabia described mild forms of ichthyosis with a thickened stratum corneum, palmar hyperlinearity and plantar keratoderma in two unrelated families with different *CLDN10* variants. Histological analysis of skin biopsies revealed slight epidermal hyperplasia and an abnormally high number of dilated eccrine sweat glands. The patients examined by Bongers et al. reported dry skin in retrospect, and dermatological consultation showed palmar hyperlinearity and plantar hyperkeratosis (personal communication with Tom Nijenhuis). In contrast, patients examined by Klar et al. and Meyers et al. showed no dermatological manifestations apart from hypohidrosis [40,41]. Morphology and number of eccrine sweat glands appeared normal in patients examined by Klar et al. [40].

### 2.3. Kidney Dysfunction

**Figure 2:**
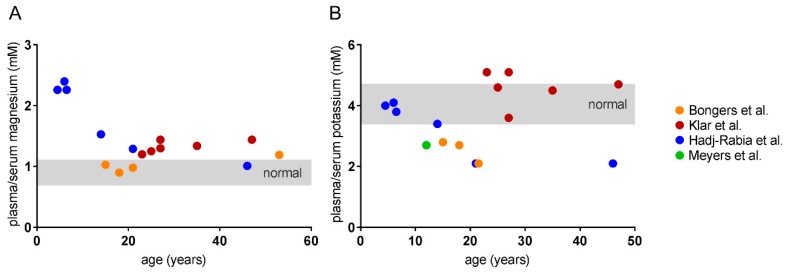
Available plasma/serum values of patients plotted against their age. (**A**) Hypermagnesemia is most pronounced in children and decreases with age. (**B**) Plasma/serum potassium levels decline with age, revealing a marked hypokalemia in adolescence. The only exception is the patient group examined by Klar et al [40]. Values were partly obtained by conversion into mmol/L. The publication by Bongers et al. provided data points of the same patients at different ages [38].

A number of features revealing a renal dysfunction in patients has been reported. All patients showed a disturbance in electrolyte balance, becoming manifest in hypermagnesemia, hypokalemia and hypocalciuria. Hypermagnesemia was present in the majority of patients, most pronounced in childhood and decreasing with age (Figure 2A). In contrast, hypokalemia was most severe in adults with the exception of the patient group examined by Klar et al. [40],(Figure 2B). These patients had serum K^+^ levels in the upper normal range or higher in adulthood. The reason for that discrepancy remains unclear. Admittedly, more longitudinal intraindividual data over a longer time period are required for confirmation of the apparent age dependencies depicted in Figure 2. Bongers et al. reported that hypokalemia was accompanied by metabolic alkalosis in their patients [38].

Of note, hypocalciuria appears to be a common manifestation as it was distinct in all tested patients and at all ages with plasma/serum Ca^2+^ in the normal or upper normal range. Despite the strong incidence of hypermagnesemia and hypocalciuria, most patients showed no decrease in urinary Mg^2+^ or an elevation of plasma/serum Ca^2+^. The reason for this is not clear. However, it appears that renal Ca^2+^ and Mg^2+^ reabsorption and handling are tightly but differentially regulated.

In some patients, plasma aldosterone levels were determined and revealed hyperaldosteronism [39,41]. The underlying cause of the electrolyte disorder and hyperaldosteronism is a NaCl wasting in the TAL, the main expression site of claudin-10b. Plasma/serum Na^+^ appeared mostly normal if tested, presumably due to compensation in the more distal parts of the nephron. However, slight hypochloremia was present in few patients [39,40,41].

Bongers et al. tested the urine concentrating ability of one patient [38]. As a result, the patient showed a reduced response to thirsting and the application of the synthetic vasopressin analogue ddAVP. Verification of an adequate aquaporin-2 response in the collecting duct pointed to a dysfunction of NaCl reabsorption in the TAL as cause for an insufficient build-up of interstitial tonicity. In line with the reduced urine concentrating ability, this patient suffered from polyuria. Overall, the occurrence of polyuria and polydipsia among patients was rather inconsistent and might also be partially attributed to xerostomia.

The estimated glomerular filtration rate (eGFR) as indicator for kidney function was determined in most patients. It ranged from low to normal and decreased with time in some patients, indicating a progressive renal insufficiency. With one exception, kidneys analyzed by computer tomography scans were normal in form and size and, in contrast to the mouse model, nephrocalcinosis was not detected in any patient [38,39,40,41]. However, several patients of Klar et al. suffered from recurrent kidney pain due to nephrolithiasis [40]. If determined, the patient’s blood pressure ranged from low to normal. Some patients complained of atypical chest pain, heart palpitations, collapse, falls, or muscle cramps. Klar et al. additionally analyzed lung and pancreas function without abnormal findings [40].

## 3. Protein Variants and Their Functional Analyses

Figure 1 displays all naturally occurring claudin-10 variants reported so far. Four mutations apply to both claudin-10a and claudin-10b proteins, whereas three defects affect only claudin-10b. Markedly, there are no obvious differences between the six patients with both defective isoforms and the 16 patients with defective claudin-10b and unaffected or only heterozygously affected claudin-10a, at least none that could be easily attributed to that special factor (Table 1). Of note, to date no patients with exclusive claudin-10a variants (without affection of claudin-10b) were described, also pointing to a minor role of claudin-10a defects in men.

Because of the predominant importance of claudin-10b defects in patients, most in vitro studies were carried out using the mutants of this isoform. Several analyses addressed localization, strand formation and channel function of mutated claudin-10b vs. wildtype protein. In the following, the denomination of claudin-10 variants refers to the alteration in claudin-10b protein, i.e., the amino acid exchange or deletion.

### 3.1. Membrane Localization

Correct trafficking to the membrane of a claudin variant is a fundamental prerequisite for a physiological function within the TJ. Similar to the wild type protein, the claudin-10b N48K mutant analyzed by Klar et al. resided in the cell membrane when heterologously expressed in the epithelial cell line MDCK C7. However, the subcellular distribution of N48K suggested an increased intracellular accumulation [40]. A very similar distribution in MDCK C7 cells was observed for the variants D73N and P149R investigated by Bongers et al. [38]. In sharp contrast, their third variant ΔE4 was very weakly expressed and did not localize to the cell membrane. This variant lacks TM4 which inevitably leads to exclusion from the plasma membrane. Likewise, the variant S131L with a missense mutation in TM3 analyzed by Hadj-Rabia et al. showed no detectable presence at the plasma membrane when expressed in a mouse TAL cell culture [39]. Contradictorily, the claudin-10 variant M1T probably lacking part of TM1 revealed normal deposition in eccrine sweat glands of a skin biopsy but was not localized in the TAL of a kidney biopsy [39]. Klar et al. also performed immunostainings of sweat glands and observed a normal localization of N48K in membranes facing the lumen but also an abnormally strong intracellular distribution without accumulation in canaliculi, pointing to a reduced delivery to the TJs [40].

Overall, the analyzed claudin-10b mutations resulted either in complete absence from the plasma membrane (especially those affecting one of the transmembrane segments) or, on the other hand, can basically insert into the plasma membrane, although an increased distribution outside the TJ suggests a reduced function.

### 3.2. TJ Strand Formation

Strand formation is the consequence of cis- and trans-interaction between claudins. In case of claudin-10b and its mutants, interaction with itself (homophilic interaction) is of particular importance as claudin-10b is not capable of interaction with any other claudin in the TAL [22]. Trans-interaction (with claudins in the opposing plasma membrane) can be detected by heterologous expression of the appropriate claudin in TJ-free HEK 293 cells and subsequent microscopic analysis of so-called contact enrichment. If the claudin is capable of autonomous strand formation, it enriches at cell–cell-contacts of two transfected cells compared to the remaining cell membrane. Bongers et al. reported a basal capability of trans-interaction and TJ formation for variants D73N and P149R but not for the truncated variant ΔE4 [38]. Klar et al. did not detect a significant contact enrichment of their mutant N48K, tantamount to a perturbed homophilic trans-interaction [40]. Moreover, homophilic cis-interaction was analyzed by Förster resonance energy transfer (FRET) and revealed an exaggerated oligomerization of N48K proteins. Ultrastructural analysis using freeze fracture electron microscopy showed that N48K formed few particle-typed TJ strands with less compact meshworks, compared to wildtype claudin-10b [40].

Overall, the few claudin-10b variants analyzed with respect to interaction properties mostly showed a fundamental capability of homophilic interaction and strand formation. Nonetheless, quality and/or quantity of interaction and strand assembly were impaired. As anticipated, the lack of a transmembrane segment resulted in a complete loss of function.

### 3.3. Channel Function

Analyses of claudin-10b mutant channel properties by means of electrophysiological measurements are available only for the N48K variant, as yet. Klar et al. stably transfected MDCK C7 cells and compared claudin-10b N48K-with wildtype-expressing cells [40]. The cation selectivity (P_Na_/P_Cl_) of N48K-expressing cells was first similar to that of claudin-10b wildtype-transfected cells but progressively decreased with passaging, more and more resembling that for clones with a weak expression of claudin-10b wildtype. Moreover, N48K-expressing cells showed a higher TER and altered relative permeabilities to other monovalent cations, compared to claudin-10 wildtype-expressing cells. Together, the results suggest that the channels formed by N48K have a subnormal Na^+^ permeability and that the overall number of channels is markedly reduced [40].

In an attempt to mimic sweat secretion, Klar et al. used a 3D cell culture model by growing MDCK C7 cells in Matrigel. Epithelial cells formed three-dimensional cysts with the apical side towards the lumen. Cysts formed by wildtype-claudin-10b-expressing cells increased their lumen by transcellular Cl^−^ secretion, followed by claudin-10b-mediated Na^+^ permeation and subsequent water transport. N48K-expressing cysts, in contrast, showed considerably less lumen expansion, indicating a reduced overall Na^+^ conductance. In line with electrophysiological studies, this is probably caused by a combination of a reduction in single channel Na^+^ conductance and a reduction in the overall number of channels [40].

### 3.4. Protein Structure

In order to determine the cause of the impairments brought about by single amino acid substitutions in claudin-10b, Klar et al. and Hadj-Rabia et al. provided 3D homology models of N48K or S131L, respectively, based on the crystal structure of murine claudin-15 [39,40,43]. The N48K mutation, localized in ECS1, appears to disrupt an intramolecular bridging between different backbones in ECS1 and membrane–ECS1-transition. Moreover, a potential electrostatic interaction could be disturbed by the replacement of an uncharged residue by a positively charged one. These alterations are presumed to perturb protein interaction and function indirectly [40]. The S131L mutation analyzed by Hadj-Rabia et al. affects TM3 and is suggested to clash sterically with several of the surrounding residues, especially of TM1 and -2 and by this perturbing the compactness of the helical bundle [39]. Furthermore, an intrahelical stabilizing hydrogen bond is lost by the amino acid substitution. As a consequence of the helical bundle destabilization, the S131L variant fails to insert into the cell membrane and is retained in the cytosol.

The impact of the amino acid substitutions D73N, R80G and P149R on protein folding or pore formation remain temporarily unsolved, also because functional data on the particular variants are scarce or absent. In general, substitution of a charged residue by an uncharged one in ECS1 (D73N, R80G) is considered critical for ion pore formation. On the other hand, neither D73 nor R80 have been shown to be important for charge selectivity of pore-forming claudins (for review see [44]). However, R80 is localized at the predicted transition between ECS1 and TM2 and might be crucial for the formation of the helical bundle. P149 in ECS2 is conserved in the majority of claudins. It is suggested to stabilize ECS2 conformation and to play a role in correct TJ strand arrangement [45].

### 3.5. Short Summary of Functional Analyses

Taken together, claudin-10b variants with truncations in one of the transmembrane segments necessarily show a complete loss of function as they are not inserted into the plasma membrane whereas variants with point mutations in one of the loops often keep a certain residual function. Nevertheless, localization, interaction and channel function can be impaired to a certain extent. Point mutations in transmembrane segments still have a high probability to severely affect membrane localization, probably depending on the substituting residue. The comparatively poor viability of cells expressing claudin-10b mutant proteins compared to wildtype-expressing cells and the increased degradation of mutant proteins due to suboptimal folding and localization as observed for several variants appear to be a common incidence and presumably contribute pivotally to the harmfulness of mutations [38,39,40].

Of course, the question arises to which extent site and type of claudin-10b mutation determine the clinical outcome. The most prominent differences between patient groups with particular *CLDN10* variants concern plasma/serum potassium values and the dermatological phenotype. However, with the actual number of patients and the information available it is difficult to assess a possible correlation and future studies will probably help to clarify that issue.

## 4. Mechanisms of Disease

**Figure 3:**
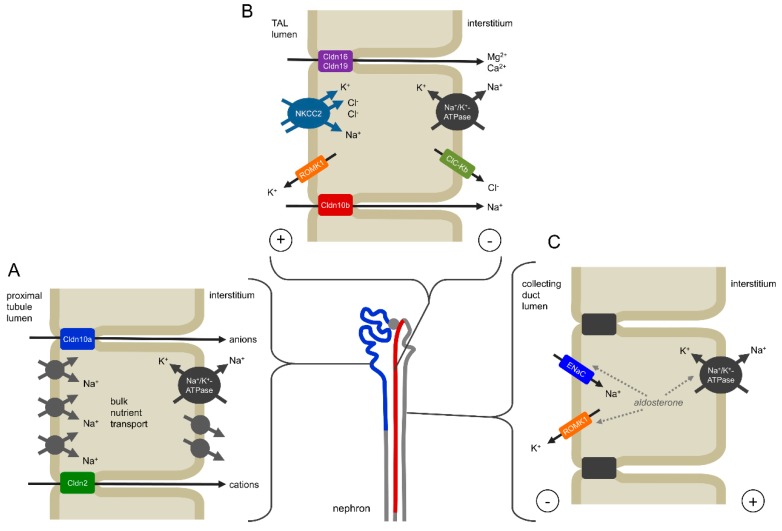
Mechanisms of claudin-10 function in the kidney. (**A**) Claudin-10a is expressed in the renal proximal tubule to the S3 segment (shown in blue) where it forms a paracellular anion pathway. Patients with pathogenic variants of both claudin-10a and claudin-10b appear to have no clinical manifestations different from patients with only claudin-10b variants, suggesting a minor role of an impaired claudin-10a function and/or compensation in more distal nephron segments. (**B**) Claudin-10b is mainly localized in the thick ascending limb of Henle’s loop (TAL, shown in red) where it facilitates the paracellular reabsorption of Na^+^. Na^+^, 2 Cl^–^ and K^+^ enter the epithelial cell by the secondary active NKCC2 transporter. Whereas Na^+^ and Cl^–^ leave the cell basolaterally, K^+^ is recirculated via the ROMK1 channel. This results in a net reabsorption of Cl^–^ and causes a lumen-positive transepithelial potential, which in turn drives paracellular Na^+^ reabsorption via claudin-10b. Towards the distal TAL segment the expression of claudin-16 and -19 is involved in the paracellular reabsorption of Mg^2+^ and Ca^2+^. Here, Na^+^ can backflux into the lumen along its concentration gradient, thus adding to the lumen-positive potential as a driving force for Mg^2+^- and Ca^2+^ reabsorption. An impairment of claudin-10b function would result in a NaCl wasting in the TAL and an expansion of claudin-16 and -19 over all TJs, causing hypermagnesemia and hypocalciuria. (**C**) Na^+^ wasting in the TAL is compensated in the more distal nephron involving electrogenic transport via ENaC, promoting secretion of K^+^ and causing hypokalemia.

**Figure 4:**
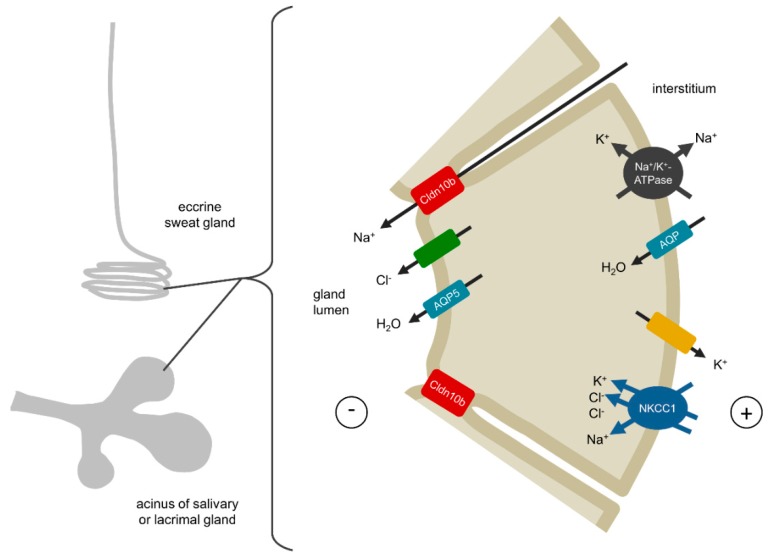
Mechanisms of claudin-10b function in the secretory portion of sweat glands and the acinar cells of salivary and lacrimal glands. During secretion, apical Cl^–^ channels and basolateral K^+^ channels are activated. Na^+^, 2 Cl^–^ and K^+^ enter the cell via the basolaterally expressed NKCC1 transporter. Cl^–^ is secreted into the lumen whereas Na^+^ and K^+^ leave the cell basolaterally. The secretion of Cl^–^ drives Na^+^ transport via the paracellular claudin-10b channel and transcellular water transport via aquaporins. An abrogation of the claudin-10b-mediated Na^+^ transport would result in a complete dysfunction of sweat, saliva and tear secretion.

The naturally occurring claudin-10 mutations discovered during the last years result in a complete or partial loss of function of the claudin-10b isoform and subsequently in a reduced or absent paracellular Na^+^ permeation in the TAL as well as in sweat, salivary and lacrimal glands. Figure 3 and Figure 4 illustrate the mechanistic principles, particularly the role of claudn-10b in epithelial transport, underlying a correct organ function.

### 4.1. Kidney

As mentioned above, the exact role of claudin-10a in the proximal tubule is not understood, as yet. Based on cell culture experiments, a function as paracellular anion channel is assumed (Figure 3A). However, the data presented in the reviewed studies indicate a rather minor role in the pathogenesis of claudin-10 defects. It appears likely that possible impairments affecting the proximal tubule can be distally compensated to a certain degree as it was shown for the loss of claudin-2 in a mouse model [46]. After all, future studies will have to clarify the physiological relevance of claudin-10a.

In the TAL, half of the Na^+^ is reabsorbed paracellularly via the claudin-10b paracellular channel as a consequence of transcellular net uptake of Cl^–^ involving NKCC2 (Figure 3B). In TAL of the ISOM, claudin-10b dominates the TJ, although claudin-3, -16 and -19 are expressed intracellularly. Towards OSOM and cortex, an additional epithelial cell type occurs, expressing claudin-3, -16 and -19 but no claudin-10b. These cells form TJ complexes that are spatially separated from claudin-10b TJs and are involved in the reabsorption of Mg^2+^ and Ca^2+^. The residual TJs assembled exclusively by claudin-10b enable a backflux of Na^+^ into the lumen, due to its concentration gradient, thus adding to the lumen-positive potential and supporting the paracellular reabsorption of Mg^2+^ and Ca^2+^ [21,22,47].

An impaired claudin-10b function would reduce the Na^+^ reabsorption in the TAL by the paracellular portion and lead to a compensatorily increased electrogenic Na^+^ reabsorption via the epithelial sodium channel (ENaC) in the more distal nephron segments. This, in turn, would promote K^+^ and H^+^ loss. Whereas hyperaldosteronism, hypokalemia and metabolic alkalosis in patients with pathogenic claudin-10b variants are the consequence of compensatory mechanisms in the distal nephron, hypermagnesemia and hypocalciuria can be attributed to an exaggerated paracellular reabsorption of Mg^2+^ and Ca^2+^ in the TAL. In the mouse model described by Breiderhoff et al., the lack of claudin-10b in the TAL results in an increased distribution of the TJ complex containing claudin-16 and -19 over all TJs, including those in the ISOM, where normally only claudin-10b is constituting the TJ [22,25,26,48]. If a similar process takes place in men with *CLDN10* variants that lead to a reduced claudin-10b insertion into TAL TJs, it would explain the hypermagnesemia, in line with the mouse model. Apparently, in patients, the reabsorption of Mg^2+^ and Ca^2+^ is shifted from cortex/OSOM TAL to ISOM TAL and the lack of claudin-10b-based Na^+^ backflux as additional driving force in cortex/OSOM TAL is thereby overcompensated.

However, the reasons for the magnitude of hypokalemia and hypermagnesemia depending on the patients’ age remain unsolved.

### 4.2. Glands

In glands, a concerted secretion of ions and water across the glandular epithelium is required in order to produce sweat, saliva or lacrimal fluid, respectively (Figure 4). Molecular mechanisms in the secretory coil of sweat glands as well as in salivary and lacrimal acinar cells are partially understood and share certain mechanistic similarities [49,50,51,52,53,54,55,56]. Stimulation of G-protein-coupled muscarinergic receptors by acetylcholine results in an increased intracellular concentration of free Ca^2+^, which activates apical Cl^−^ channels and basolateral K^+^ channels. Subsequent activation of NKCC1 in the basolateral membrane leads to an influx of Na^+^, K^+^ and Cl^–^ into secretory cells. Na^+^ and K^+^ circulate across the basolateral membrane via NKCC1, the Na^+^/K^+^-ATPase and/or K^+^ channels, respectively. The transcellular net flux of Cl^−^ via apical channels and basolateral NKCC1 generates a lumen-negative transepithelial potential and drives the paracellular secretion of Na^+^ via claudin-10b channels. This is followed by transcellular water secretion involving aquaporin-5 water channels. Although an additional paracellular water secretion is frequently suggested, the strict water impermeability of claudin-10b-based TJs [13] and the implausibility of claudin-10b to form heterogeneous TJs with other claudins [22] rather argue against it.

An impairment of claudin-10b distribution or function as reported by Klar et al. would result in a complete abrogation of Na^+^ and subsequent water secretion in sweat, salivary and lacrimal glands and become manifest in hypohidrosis, aptyalism and alacrima as observed in patients [40].

### 4.3. Skin

At present it remains unsolved whether dermatological manifestations such as ichthyosis and dry skin are a mere consequence of sweat gland dysfunction or may also be augmented by defective claudin-10 proteins in the epidermis. The mechanical barrier function of the skin is maintained on the one hand by the stratum corneum containing dead, cornified cells (corneocytes) and by the TJs on the other hand (for review see [57]). Functional TJs are localized in the stratum granulosum where they form a liquid–liquid interface barrier. The role of claudins in epidermal barrier function is illustrated by the phenotype of patients with pathogenic variants of the *CLDN1* gene. Patients lacking functional claudin-1 suffer from NISCH syndrome (neonatal ichthyosis–sclerosing cholangitis). The thick, scaly skin of ichthyosis is assumed to result from the skin compensating for barrier dysfunction [58]. On the other hand, claudins are expressed not only in the stratum granulosum but in all living layers of the epidermis and are probably involved in the differentiation of stratum granulosum cells to corneocytes.

Claudin-10 mRNA was detected in human skin biopsies and was found to be downregulated in patients with psoriasis vulgaris [59,60,61]. Immunohistochemical staining of rodent skin revealed an intracellular localization in the stratum basale [20] or in the stratum corneum [62]. The finding that claudin-10 is expressed beyond the stratum granulosum points to a possible function aside from its role as an ion pathway in the epidermis. However, its particular function in the skin has yet to be unraveled.

## 5. Summary

The four studies of Bongers et al., Klar et al., Hadj-Rabia et al. and Meyers et al. describe a novel claudinopathy that is based on mutations in the *CLDN10* gene and is characterized by an impaired function of mainly claudin-10b. Patients show a salt-losing nephropathy without hypercalciuria (as in Bartter syndrome) or hypomagnesemia (as in Gitelman syndrome). Main symptoms probably occurring in the majority of present and future patients are:

- age-dependent hypermagnesemia, age-dependent hypokalemia, hypocalciuria;
- hypohidrosis, xerostomia, alacrima;
- possibly dermatological abnormalities.

The severity of clinical manifestations may depend on site and type of mutation. Patients expressing a claudin-10b variant with a defect in one of the transmembrane regions, especially a truncation, are at high risk to develop the full severity of the phenotype. In this respect, genotyping of future patients may further help to correlate mutation type and course of disease. Further functional analysis of found *CLDN10* variants may provide a more detailed insight into the protein function. The reviewed studies demonstrate the significance of proper claudin-10b function for kidney, skin and gland physiology.

## Figures and Tables

**Table 1:** Summary of all patient groups, their *CLDN10* variants and clinical manifestations.

Publication	Bongers et al. [38]	Bongers et al. [38]	Klar et al. [40]	Hadj-Rabia et al. [39]	Hadj-Rabia et al. [39]	Meyers et al. [41]
Number of patients	1 (patient 1)	1 (patient 2)	13	4 (family A)	2 (family B)	1
Claudin-10a variant	P147R, ΔE4	wildtype, P147R	wildtype	S129L	wildtype	R78G
Claudin-10b variant	P149R, ΔE4	D73N, P149R	N48K	S131L	M1T	R80G
Loss of claudin-10b function	partial/complete	partial	partial	complete	complete *	not analyzed
Extrarenal manifestations						
Xerostomia	yes	yes	yes	yes	yes	yes
Alacrima	n.r.	yes	yes	yes	yes	yes
Hypohidrosis	yes	yes	yes	yes	yes	yes
Dermatological manifestations in addition to hypohidrosis	yes	yes	no	yes	yes	no
Renal function						
Hypermagnesemia	yes	no	yes	yes	yes **	yes
Urinary magnesium	rather high ***	normal ***	low	low or normal	rather high	n.r.
Plasma/serum calcium	upper normal range	upper normal range	normal	1 increased, 3 normal	normal	n.r.
Hypocalciuria	yes	yes	yes	yes	yes	yes
Hypokalemia	yes	yes	no	yes **	yes **	yes
Metabolic alkalosis	yes	yes	n.r.	n.r.	n.r.	n.r.
Plasma aldosterone	n.r.	n.r.	n.r.	normal or high	normal or high	high
Polyuria	yes	no	no	inconsistent	inconsistent	yes
Polydipsia	n.r.	n.r.	n.r.	yes	yes	yes
Estimated Estimated glomerular filtration rate	decreased	normal	lower normal range	normal or decreased	normal	decreased
Kidney form/size	small right kidney	normal	normal	n.r.	normal	normal
Nephrocalcinosis	no	no	n.r.	no	no	no
Blood pressure	lower normal range	low to normal	n.r.	low	low	normal

* with the exception of normal deposition in eccrine sweat glands. ** considering the patient’s age. *** compared to heterozygous family members. n.r. not reported
